# Modifying Commercial Poly(ethylene-vinyl acetate) to Improve Its Performance as a Pour Point Depressant: A Study in Waxy Model Oils at Different Concentrations

**DOI:** 10.1021/acsomega.5c03230

**Published:** 2025-09-15

**Authors:** Maximiliano F. Martins, Ingrid Vitória J. B. de Sousa, Victor Augusto M. C. da Silva, Pedro Victor R. Severo, Bruna F. Alves, Rita de Cássia P. Nunes, Dellyo R. S. Alvares, Elizabete F. Lucas

**Affiliations:** † Metallurgical and Materials Engineering Program, COPPE/LADPOL, Federal University of Rio de Janeiro, Av. Horácio Macedo, 2030, Bloco F, 21941598 Rio de Janeiro, Rio de Janeiro, Brazil; ‡ Macromolecules Institute, Federal University of Rio de Janeiro, Rua Moniz Aragão, 360, Bloco 8G/CT2, 21941594 Rio de Janeiro, Rio de Janeiro, Brazil; § Leopoldo Américo Miguez de Mello Research, Development and Innovation Center (CENPES), 42506 Petrobras, Av. Horácio de Macedo, 950, 21941915 Rio de Janeiro, Rio de Janeiro, Brazil

## Abstract

Poly­(ethylene-vinyl acetate) (EVA) is the most used pour point depressant (PPD) in crude oil. Because it does not exhibit a high performance in all kinds of oils, other structures have been studied. Both hydrolyzed EVA and EVA:clay minerals can exhibit a higher performance than unmodified ones, but their mechanisms of action have not yet been fully clarified. In this work, we evaluated for the first time a nanocomposite based on EVA joining the contribution of hydrolysis and clay minerals, besides the proposition of action mechanisms. For this purpose, two EVA commercial samples (EVA7 and EVA11 containing 7.0 and 11.0 mol % vinyl acetate, respectively) were hydrolyzed, and EVAOH:palygorskite nanocomposites were prepared. The products were characterized by nuclear magnetic resonance, energy-dispersive spectroscopy, and scanning and transmission electron microscopy. Their behavior was evaluated in terms of pour point and morphology by optical microscopy, using waxy model oils with different wax contents in toluene. Both unmodified EVA and hydrolyzed EVA7 exhibited the worst performance. In contrast, EVA11:PALY, EVA11OH, and EVA11OH:PALY outperformed EVA11. However, the combined effect of hydrolysis and nanoclay in the EVA11OH:PALY nanocomposite did not significantly improve its performance. This behavior suggests that the effect of the hydroxyl groups overlapped that of the clay mineral. Optical microscopy images revealed that the additives produced different wax crystal morphologies, which is directly related to the performance. Moreover, the spacing between crystals plays a more important role in oil fluidity than the reduction in the crystal size. A correlation between the polarity and solubility of the additives in the model system and their action mechanisms could be proposed.

## Graphical abstract

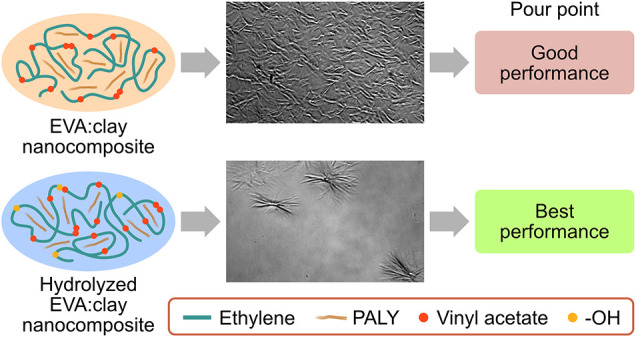

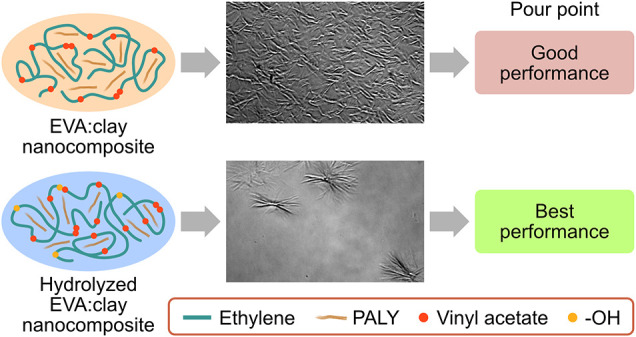

## 1 Introduction

The application of polymeric materials as flow improvers has grown in recent years in response to new challenges faced by the petroleum industry. The need to facilitate the flow of systems containing high-molecular-weight paraffins drives the development of pour point depressant (PPD) additives.^1–6^

For a PPD to be effective, it is important for its chemical structure to contain nonpolar hydrocarbon chains and polar groups so that it is able to cocrystallize with paraffins and sterically prevent the formation of a crystalline network. In some cases, the additive can even hinder the precipitation of paraffins at lower temperatures.^1,7–9^ Among the polymeric materials used for this purpose, the literature describes polymers based on esters, vinyl acetate, maleic anhydride, acrylates, methacrylates, and/or acrylonitriles. The polar groups present in these materials favor the morphological alteration of the crystals, helping to inhibit the formation of large three-dimensional paraffinic networks.^10–17^ Poly­(ethylene-vinyl acetate) (EVA) and its derivatives constitute one of the most investigated classes of materials to flow assurance of waxy oil systems, due to their good performance as PPD and the potential for improving their properties through structural modifications in the polymer.^4,6,18–20^

Polymeric composites and nanocomposites also stand out as PPDs and flow improvers, because fillers, such as polymer, graphene oxides, carbon nanotubes and clay minerals, when incorporated into the polymer matrix, can effectively influence the wax crystallization process.^21–28^ Palygorskite (PALY) is a clay mineral with a fibrillar morphology that exhibits excellent physical and chemical properties.^29,30^ Its fibers have diameters on the nanometric scale, a key feature for its use as a filler in the production of nanocomposite materials.^31^ Due to the presence of surface charges in its structure, the preparation of polymer composites incorporating this clay mineral often requires the use of an ionic surfactant, which acts by increasing the lipophilicity of the mineral, allowing its compatibility with the polymer matrix.^29,32^ Although this class of materials has potential for application as PPD, the understanding of how the mechanism of action of nanoparticles affects their performance is still limited.^33^

Studies involving the modification of EVA through hydrolysis reactions have shown that increasing the polarity of the material, up to a certain limit, contributes to increasing the efficiency of the additive as PPD.^4,34^ The literature also indicates that nanocomposites based on EVA:PALY outperform EVA in reducing pour point (PP) in waxy model oils.^27^ Thus, we investigated for the first time a polymer nanocomposite formulated with a hydrolyzed EVA matrix and PALY as a filler. We compared the performance of this material with that of a nanocomposite of unmodified EVA and PALY, as well as samples of unmodified EVA and hydrolyzed EVA, to assess their performances in PP tests and crystal morphology analyses using waxy model oils composed of commercial paraffin in toluene at varying concentrations.

## 2 Experimental Section

### 2.1 Materials

Two EVA samples were supplied by Braskem S.A. (São Paulo, Brazil), with different vinyl acetate (VA) contents: 6.9 and 11.2 mol %, called, respectively, EVA7 and EVA11. Hydrochloric acid (HCl) 37% with PA purity grade, sodium hydroxide (NaOH) with PA purity grade, and methanol (MeOH) with >99% purity were supplied by Isofar Indústria e Comércio de Produtos Químicos Ltd.a. (Rio de Janeiro, Brazil). Deuterated chloroform (CDCl_3_) with 99.8% purity, cetyltrimethylammonium bromide (CTAB) with 99% purity, Formvar/carbon-supported copper grids with a grid size of 300 mesh (model FCF300-CU), a Spurr low-viscosity resin embedding kit (to graft the polymer for TEM analysis), the clay mineral PALY utilized to produce the polymeric nanocomposites (from the Coimbra mine, Piau State, Brazil), and tetrahydrofuran (THF) with ≥99.0% purity, all manufactured by Sigma-Aldrich, were supplied by Merck Brazil (São Paulo, Brazil). Commercial-grade toluene, used after being distilled and dried on alumina at room temperature, and the wax (melting point in the range 56–58 °C), containing *n*-paraffins ranging in the size from C18 to C44 with a predominance of C28,^34^ were provided by Vetec Quimica Fina Ltd.a. (Rio de Janeiro, Brazil).

### 2.2 Production of the Materials

#### 2.2.1 Chemical Modification of EVA Samples

Samples of EVA7 and EVA11 were subjected to hydrolysis reactions of the VA groups, with theoretical modification degrees of 2.0, 4.0, 6.0, and 8.0 mol %. Modification degrees lower than 10.0 mol % were selected to avoid an excessive increase in the polarity of the materials, which could compromise their solubility in toluene.^4,34^ In the specific case of EVA11, this precaution was even more relevant because the low solubility of the matrix could hinder the incorporation of the clay mineral during the composite preparation. Among the different modification degrees, only the modified materials that showed a significant performance in the PP tests were included in this work: EVA7, with a theoretical hydrolysis degree of 2.0 mol %, and EVA11, with a theoretical hydrolysis degree of 6.0 mol %. These samples were called EVA7OH and EVA11OH, respectively.

The chemical modification of the materials followed the method described by the literature.^35^ EVA in toluene was reacted with a methanolic solution of sodium hydroxide (NaOH) at 10 wt/v % at 110 °C for 2 h. The reaction product was precipitated using an aqueous solution of hydrochloric acid (HCl) at 20% v/v, filtered, and dried in a vacuum oven. Table 1 presents the parameters used in the hydrolysis reactions of the EVA samples.

**Table 1:** Parameters Used for EVA Hydrolysis^a^

modified sample code	origin sample	VA mol (n)	theoretical hydrolysis degree (%)	NaOH mol (n)	mass of methanolic NaOH solution (g)
EVA7OH	EVA7	0.0221	2	4.41 × 10^–4^	0.1765
EVA11OH	EVA11	0.0325	6	1.95 × 10^–3^	0.7804

a VA = vinyl acetate.

#### 2.2.2 Preparation of Nanocomposite Materials

Two composite materials were prepared using EVA11 and EVA11OH samples as matrices and the clay mineral PALY as the filler. The matrix/filler ratio used in both composites was 95:5.^27^ Before use, the clay mineral was processed through comminution, carried out by wet grinding in an Essa jaw crusher (model 185020) for 1 min, followed by the filtration of the suspension and subsequent drying of the sample. After being dried, the material was subjected to magnetic separation to remove metallic compounds present in the mineral. The processed clay mineral was organically modified with the surfactant CTAB to promote its compatibility with the polymer matrix.^29^ The composites were prepared by a solution and solvent precipitation method. A dispersion of PALY and polymer (EVA or hydrolyzed EVA) in THF was subjected to an ultrasonic bath for 1 h, followed by mechanical stirring at 80 °C until the complete evaporation of the solvent. The resulting film was placed in a desiccator and maintained under reduced pressure for 48 h until complete drying.

### 2.3 Characterization of the Materials

#### 2.3.1 Nuclear Magnetic Resonance

The hydrolyzed EVA samples were characterized by hydrogen nuclear magnetic resonance (^1^H NMR) spectroscopy to determine the degree of modification achieved. The analyses were conducted with a Bruker NMR spectrometer (model Avance III 500 MHz). The samples were prepared at a concentration of 45 mg/mL, using CDCl_3_ as the solvent and tetramethylsilane (TMS) as the internal standard. The spectra were obtained with an acquisition time (d1) of 1 s, pulse duration (p1) of 10700 μs, and spectral width (SWH) of 10 kHz.

#### 2.3.2 Scanning Electron Microscopy and Energy-Dispersive Spectroscopy

SEM was used to examine the morphology of the composite materials, while EDS analysis allowed the identification and quantification of the chemical elements present in the samples. The analyses were performed with a Tescan VEGA3 LMU microscope, operating at 10 kV, coupled to an EDS analyzer with an acceleration voltage of 20 kV, operating with a working distance of 15 mm, and a current intensity of 14 nA. For the analyses, the samples were comminuted in an IKA A11 basic analytical mill, followed by carbon coating via cathodic sputtering using a Denton Vacuum Desk V metalizer.

#### 2.3.3 Transmission Electron Microscopy

The aggregation state of the clay mineral fibers in the polymer matrix was evaluated by using the TEM technique. The analyses were conducted with a Thermo Fisher Scientific Talos F200X microscope, operating with a current intensity of 200 kV. To perform these analyses, the samples were embedded in molds using embedding resin and cured in a forced-air oven at 70 °C for 24 h. Subsequently, the samples were sectioned by using a Leica EM UC7 ultramicrotome with cutting intervals between 40 and 70 nm. After sectioning, the samples were supported on TEM grids.

### 2.4 Preparation of the Waxy Model Oils

The waxy model oils were prepared at concentrations of 3.0, 6.0, and 9.0 wt/wt % by dissolving commercial paraffin in toluene, followed by magnetic stirring and heating at 45 °C for 1.5 h. The additives were prepared by dissolving the materials in toluene at 10.0 wt/wt % and subjecting the systems to stirring and heating at 60 °C for 1.5 h. For the incorporation of the additives in the model oil, each preprepared additive sample in toluene was weighed in a beaker, using the required mass to achieve the desired active substance concentrations: 500, 1000, or 2000 ppm (which corresponds, respectively, to 4500, 9000, or 18000 ppm of toluene in the system). Immediately afterward, the previously heated model oil was weighed in the same container. The systems with additives were then subjected to heating and stirring at 45 °C for 1 h and then left to rest for 24 h. Additional tests were performed with the addition of 20,000 ppm of pure toluene to the waxy model systems, and no reduction in PP was observed. Therefore, these results were not presented in the manuscript.

### 2.5 Evaluation of Waxy Model Oils

The pure waxy model oil and the systems with different additives at different concentrations were evaluated by PP tests and polarized optical microscopy.

#### 2.5.1 PP Test

PP tests were used to determine the temperature at which samples stopped flowing under the action of gravity. The tests were based on ASTM D97, which consists of a manual method of measuring the temperature using a thermometer inserted into the sample and a sequence of thermostatic baths set at temperatures of 24.0, 0, −18.0, and −33.0 °C, used to gradually reduce the sample temperature.^36^ Analyses were performed in duplicate with a maximum standard deviation of ±1.5 °C.

#### 2.5.2 Polarized Optical Microscopy

POM analyses were performed to identify the morphology of wax crystals in the additivized systems. The samples were analyzed using an Axio-Zeiss Axiocam 705 color optical microscope coupled to a Linkam temperature control system, and the images were observed under transmitted light, with polarization and 20× magnification. The analyses were conducted using the following program: (i) heating range to 45 °C at 20 °C/min followed by (ii) cooling range from 45 to 5 °C at 2 °C/min. Micrographs were captured at temperatures of 5 and 15 °C.

## 3 Results and Discussion

### 3.1 Hydrolysis Degree of the Modified EVA Samples

The determination of the hydrolysis degree obtained in the chemical modification of the EVA samples was performed through ^1^H NMR analyses, by integrating the signals corresponding to the proton directly bonded to the carbon of the –OH group and the protons of the CH_3_ group of VA, centered at 3.59 and 2.03 ppm, respectively. The calculations were carried out using eq 1

1 $$\%\;hydrolysis=\frac{3\times A_{3.64-3.54\;ppm}}{\left(3\times A_{3.64-3.54\;ppm}\right)+A_{2.08-1.98\;ppm}}\times 100$$

where *A*_3.64–3.54 ppm_ corresponds to the integration of the area under the signal centered at 3.59 ppm and *A*_2.08–1.98 ppm_ corresponds to the integration of the area under the signal centered at 2.03 ppm.

Figure 1 shows the ^1^H NMR spectra of the EVA7OH and EVA11OH samples and the chemical structure of hydrolyzed EVA, highlighting the signals that were used to calculate the hydrolysis degree. Table 2 presents the calculated values for the hydrolysis degrees of the modified EVA samples.

**Figure 1:**
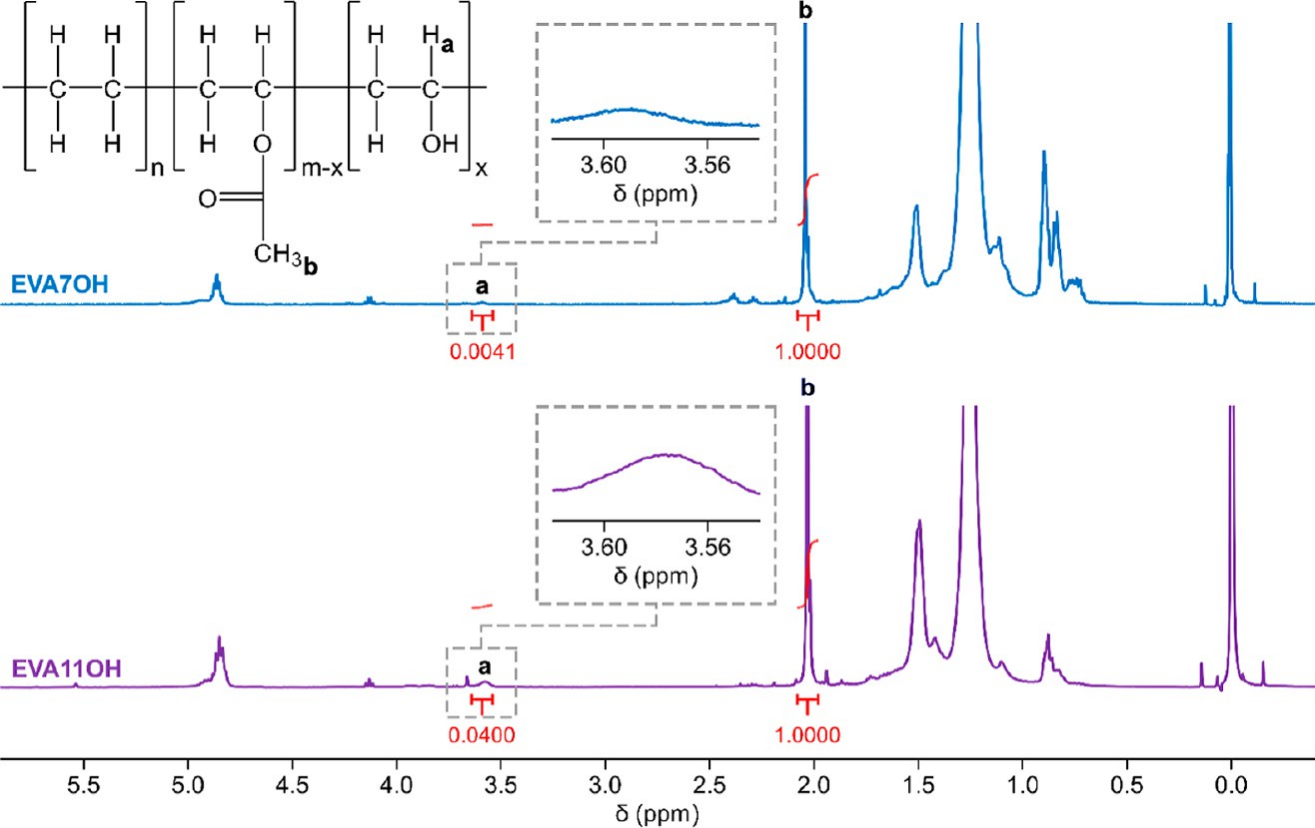
^1^H NMR spectra in CDCl_3_ of EVA7OH and EVA11OH.

**Table 2:** Degree of Hydrolysis Determined for the Modified EVA Samples

material code	theoretical hydrolysis degree (mol %)	*A*_3.64–3.54 ppm_	*A*_2.08–1.98 ppm_	experimental hydrolysis degree (mol %)
EVA7OH	2	0.0041	1.0000	1.22
EVA11OH	6	0.0400	1.0000	10.71

None of the reactions produced the theoretical hydrolysis degree initially predicted, resulting in modification percentages of 1.22% and 10.71% for EVA7OH and EVA11OH, respectively. This result demonstrated the difficulty of controlling this type of reaction, possibly due to weighing errors, solvent evaporation, and other process variables.

### 3.2 Electronic Microscopy and EDS Analysis

Figure 2 shows SEM micrographs of the EVA11:PALY and EVA11OH:PALY composites and their respective matrices.

**Figure 2:**
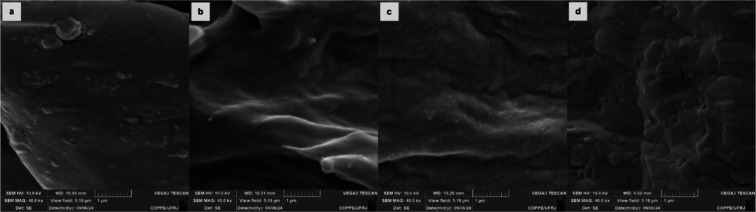
SEM micrographs of (a) EVA11; (b) EVA11OH; (c) EVA11:PALY; and (d) EVA11OH:PALY.

Figure 2a,b presents the SEM micrographs of the EVA11 and EVA11OH samples, respectively, revealing a difference in the morphology of the materials: a smoother surface for EVA11 and a rougher surface for EVA11OH. This behavior may be related to a better organization of the polymer chains in the solid state in the commercial EVA. In contrast, in EVA11OH, the increase in polarity resulting from hydrolysis may have hindered the reorganization of the polymer chains, possibly due to a greater repulsion between these chains. The samples of the EVA11:PALY and EVA11OH:PALY composites (Figure 2c,d), in addition to having distinct morphologies in relation to their respective matrices (Figure 2a,b), also exhibited morphological differences between them, more pronounced in comparison with the polymer matrices. This effect can be explained by the presence of a clay mineral in the composition of the composites, further increasing the polarity of the materials.

Despite the morphological differences observed, at the magnification achieved in the analyses (40,000×), it was not possible to identify phase separation on the surface of the materials, indicating that the clay mineral load was dispersed in the polymer matrix. Based on these results, we performed EDS analyses to investigate the incorporation of the clay mineral into the matrix. Figure 3 shows the EDS spectra of samples EVA11, EVA11OH, EVA11:PALY, and EVA11OH:PALY.

**Figure 3:**
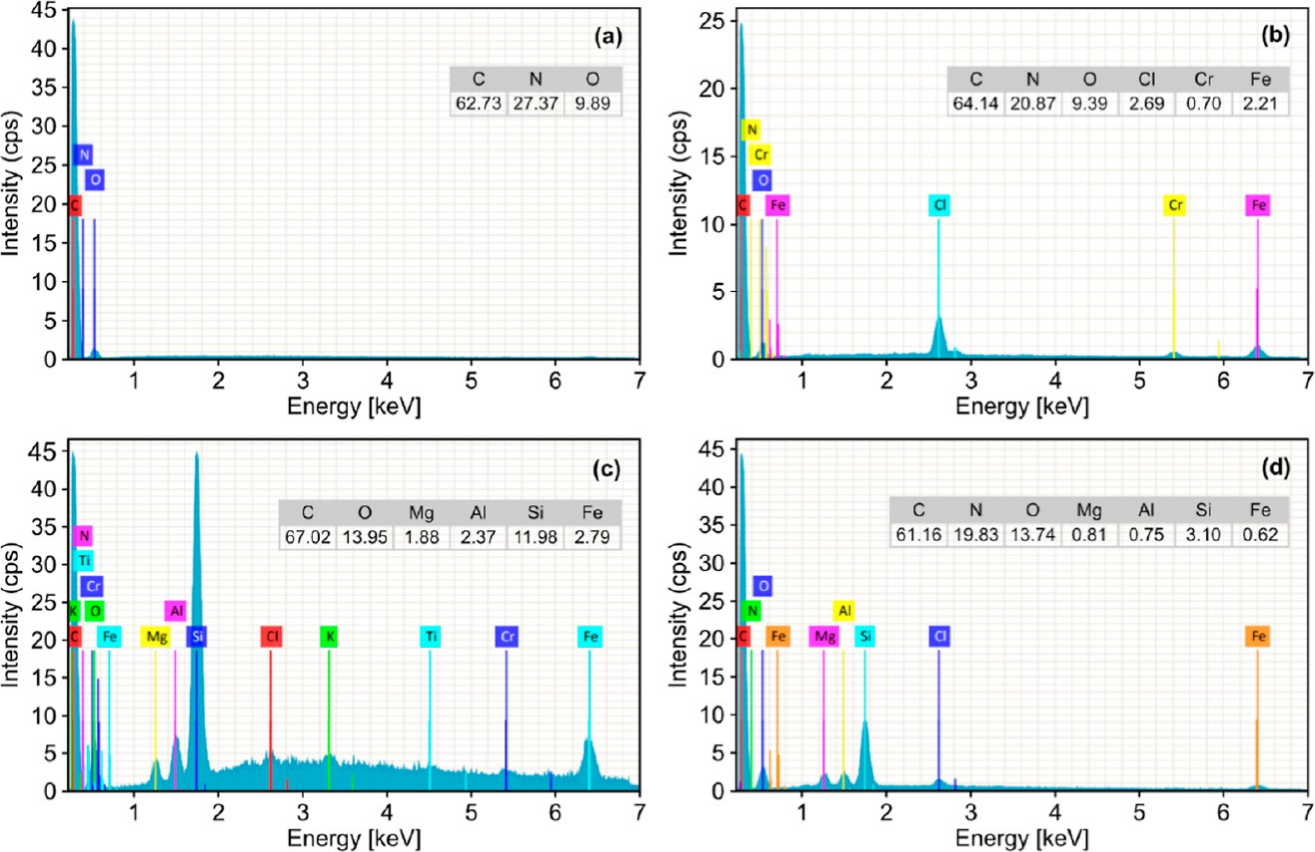
EDS spectra of (a) EVA11; (b) EVA11OH; (c) EVA11:PALY; and (d) EVA11OH:PALY.

In the spectrum of commercial EVA11 (Figure 3a), peaks corresponding to C and O are mainly observed, referring to the polymer composition. In contrast, in the hydrolyzed EVA11 sample (Figure 3b), additional peaks are observed, related to the presence of Cr, Fe, and Cl in the material composition. The presence of these elements was probably associated with a small amount of impurities generated during the chemical modification stages and the material comminution process. In the spectra of the composites (Figure 3c,d), peaks corresponding to Al, Fe, Mg, and Si can be observed, which are characteristic elements of the clay mineral PALY.^27,37^ The presence of these peaks in the spectra of both materials confirmed the incorporation of the clay mineral into the polymer matrix and successful formation of the composites.

Figure 4 shows the TEM micrographs of EVA11:PALY and EVA11OH:PALY. This analysis revealed that in both samples, the clay mineral fibers were completely disaggregated and dispersed in the polymer matrix. Furthermore, the micrographs revealed that the fibers had regions of different thicknesses with diameters varying between 70 and 100 nm, characterizing the formation of nanocomposite materials.

**Figure 4:**
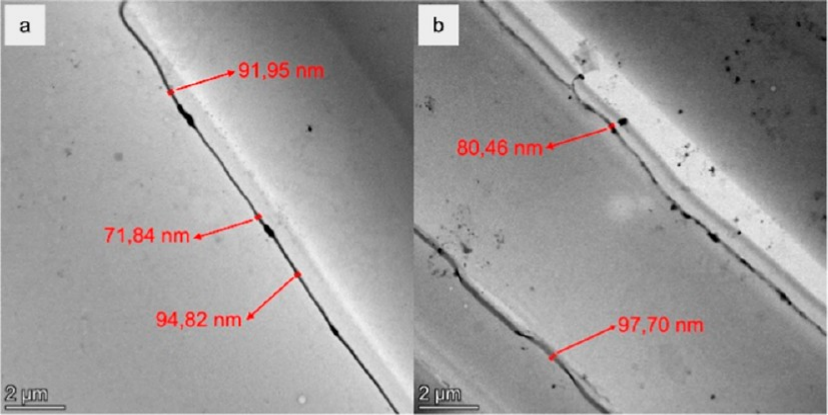
TEM micrographs of (a) EVA11:PALY and (b) EVA11OH:PALY.

### 3.3 Pour Point

The performance evaluation results of all materials carried out through PP tests are presented in Table 3. All materials that showed some reduction in PP in the model oil at 3.0 wt/wt % paraffin were analyzed at the highest concentrations (6.0 or 9.0 wt/wt %) until the additive efficiency was no longer observed. In relation to unmodified EVA samples, EVA7, containing 7.0 mol % VA, was able to reduce the PP of the model oil with 3.0 wt/wt % paraffin from 15 to 1.5 °C at a concentration of 2000 ppm. In contrast, EVA11, containing 11 mol % VA, was more efficient, being able to reduce the PP of the model oil at the same paraffin concentration to <−24.0 °C at all additive concentrations evaluated. This result agrees with the literature that indicates an optimal VA content for EVA to achieve the best efficiency in reducing the pour point.^38^

**Table 3:** Pour Point of Pure and Added Waxy Model Oils^a^

		wax concentration (wt/wt %)					
		3.0		6.0		9.0	
material code	*C*_AS_ (ppm)	PP (°C)	Δ_PP_ (°C)	PP (°C)	Δ_PP_ (°C)	PP (°C)	Δ_PP_ (°C)
no additive		15.0		18.0		24.0	
EVA7	500	6.0	–9.0	18.0	0		
	1000	7.5	–7.5	16.5	–1.5		
	2000	1.5	–13.5	16.5	–1.5		
EVA7OH	500	10.5	–3.0	18.0	0		
	1000	4.5	–10.5	15.0	–3.0		
	2000	4.5	–10.5	15.0	–3.0		
EVA11	500	<−24.0	>−39.0	9.0	–9.0	21.0	–3.0
	1000	<−24.0	>−39.0	15.0	–3.0	24.0	0.0
	2000	<−24.0	>−39.0	15.0	–3.0	24.0	0.0
EVA11OH	500	<−24.0	>−39.0	<−24.0	>−42.0	10.5	–13.5
	1000	<−24.0	>−39.0	–12.0	–30.0	21.0	–3.0
	2000	<−24.0	>−39.0	15.0	–3.0	21.0	–3.0
EVA11:PALY	500	<−24.0	>−39.0	–15.0	–33.0	24.0	0
	1000	<−24.0	>−39.0	9.0	–9.0	24.0	0
	2000	<−24.0	>−39.0	18.0	0	27.0	3.0
EVA11OH:PALY	500	<−24.0	>−39.0	<−24.0	>−42.0	6.0	–18.0
	1000	<−24.0	>−39.0	<−24.0	>−42.0	18.0	–6.0
	2000	<−24.0	>−39.0	–1.5	–19.5	18.0	–6.0

a *C*_AS_ = active substance concentration; Δ_PP_ = [PP_added system_ – PP_pure system_]; maximum PP error = 1.5 °C.

When the paraffin concentration was increased to 6.0 wt/wt %, EVA7 was no longer able to reduce the pour point at any of the concentrations tested. EVA11, in turn, was able to reduce the pour point by 9.0 °C only at a concentration of 500 ppm, with its efficiency decreasing thereafter with the increase in the additive concentration. This result justified the analysis of EVA11 in model oils containing paraffins at 9.0 wt/wt %. However, no efficiency was observed in the systems with a higher concentration of paraffin.

To evaluate the influence of increased polarity in the two EVA samples, we partially hydrolyzed them. EVA7OH exhibited a performance very close to that of its base material in model oils at 3.0 and 6.0 wt/wt % paraffin and was not evaluated in systems with the highest paraffin concentration. This behavior indicated that increasing only the relative polarity of the molecules, without increasing the number of polar groups, did not affect the action of the additive. On the other hand, EVA11OH was more efficient than EVA11 in the system at 6.0 wt/wt % paraffin at concentrations of 500 ppm (from Δ_PP_ = −9.0 to >−42.0 °C) and 1000 ppm (from Δ_PP_ = −3.0 to −30.0 °C). This result justified the evaluation of this material in the model system containing 9.0 wt/wt % paraffin, where a good efficiency (Δ_PP_ = −13.5 °C) was observed only at 500 ppm. This behavior confirmed the improvement in the efficiency with the increase in the polarity of the molecules in materials with an optimal VA content, within its solubility limit in the organic environment in question.^4,39^

Samples, EVA11 and EVA11OH, unlike the EVA7 samples, showed a better performance at the lowest additive concentrations tested. This phenomenon could be related to the polymer’s hydrodynamic volume. That is, the reduction in the molecules’ hydrophobicity promoted greater solubility in toluene, expanding the hydrodynamic volume in solution. As a result, the ethylene segments, which are capable of interacting with paraffins, became more available, thereby promoting a greater reduction in the PP observed for these materials. In contrast, the expansion of the polymer coil reduced the intermolecular distance between the species at higher concentrations of the material in the system, thereby promoting a greater polymer–polymer interaction, resulting in the loss of efficiency observed.

Regarding the systems containing 3.0 wt/wt % paraffin, EVA11, EVA11OH, EVA11:PALY, and EVA11OH:PALY were able to reduce the PP to <−24 °C at all additive concentrations. At a concentration of 6.0 wt/wt % paraffin, EVA11:PALY was more efficient than its base material at concentrations of 500 and 1000 ppm, and like EVA11, it was unable to reduce the pour point at a concentration of 2000 ppm, confirming what has already been reported in the literature.^27^ EVA11OH:PALY outperformed its base material at concentrations of 1000 and 2000 ppm, showing a similar efficiency as that at a concentration of 500 ppm. In systems containing 9.0 wt/wt % paraffin, both EVA11:PALY and its base material were not able to reduce the pour point at any of the additive concentrations evaluated. On the other hand, EVA11OH and EVA11OH:PALY showed a good efficiency but only at a concentration of 500 ppm, at which both had a similar performance.

The presence of PALY in the polymer matrix was capable of improving the efficiency of both modified and unmodified EVA (as observed in the systems at 6.0 wt/wt % paraffin). However, in the tests with 9.0 wt/wt % paraffin, for the EVA11OH:PALY sample, the effect of the –OH group on the matrix structure outweighed the influence of the clay mineral load. This behavior could be related to the greater availability of polar groups (−OH) along the polymer chain compared with the occluded charges on the surface of the mineral bonded to the polymer matrix, as will be discussed in Section 3.5.

### 3.4 Polarized Optical Microscopy

POM analyses were performed for the system containing 6.0 wt/wt % of paraffin without and with 2000 ppm of the active substance. The materials that exhibited PP reduction capacity at this paraffin concentration were also evaluated in systems containing 500 ppm of the active substance, both at the 6.0 wt/wt % paraffin and at the highest concentration of 9.0 wt/wt %. The micrographs of the systems contained 2000 ppm of EVA (EVA7, EVA7OH, EVA11, and EVA11OH) and nanocomposites (EVA11:PALY and EVA11OH:PALY) at 15 and 5 °C are shown in Figure 5.

**Figure 5:**
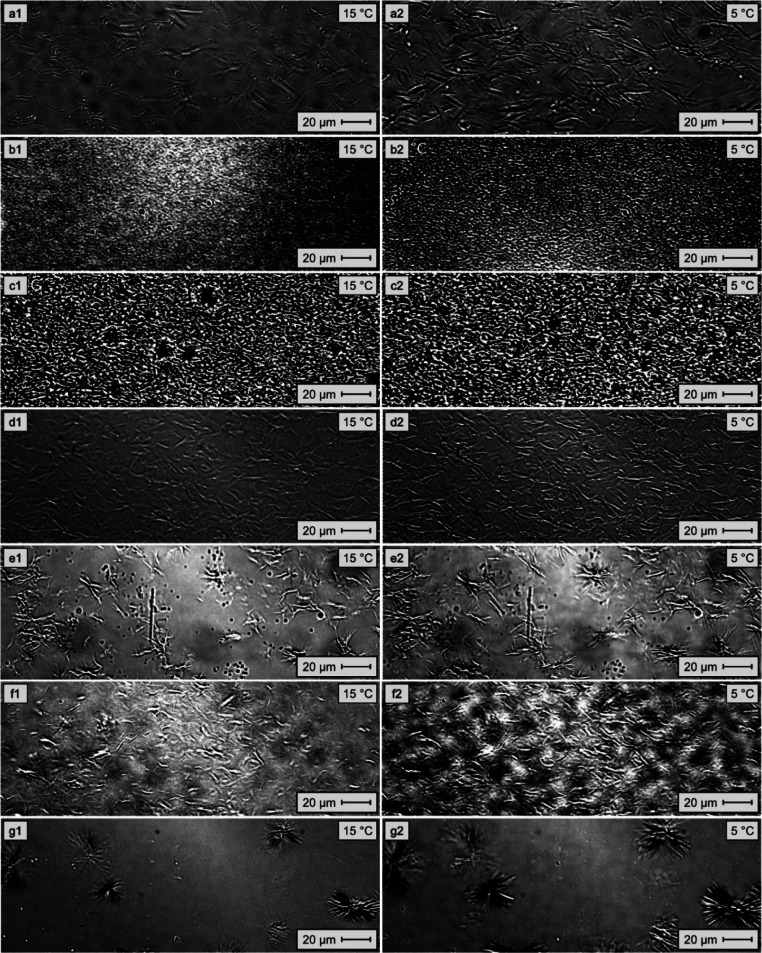
Micrographs of model oils with 6.0 wt/wt % paraffin in toluene, pure and mixed with 2000 ppm of the active substance at 15 and 5 °C, respectively: (a1,a2) no additive; (b1,b2) EVA7; (c1,c2) EVA7OH; (d1,d2) EVA11; (e1,e2) EVA11OH; (f1,f2) EVA11:PALY and (g1,g2) EVA11OH:PALY.

All materials (Figure 5b–g) modified the crystalline morphology of the pure system containing 6.0 wt/wt % paraffin (Figure 5a), reducing, to a greater or lesser extent, the size of the crystals and producing different morphological patterns.

The systems containing EVA7 and EVA7OH already presented a dense and compact crystalline network at a temperature of 15 °C (Figure 5b1,c1, respectively) with voids surrounded by wax agglomerates. The dense and compact morphology of the waxes is consistent with the inefficiency of these two materials in reducing the PP.

In contrast, EVA11-based samples exhibited a less dense morphology with crystals further apart. The comparison of the influence of EVA11 and EVA11OH on the model oil revealed a change in the crystal morphology at both 15 and 5 °C: The crystals changed from long needle-like structures with EVA11 (Figure 5d1,d2) to a star-shaped arrangement and greater spacing between the crystals with EVA11OH (Figure 5e1,e2). This behavior was likely related to the increase in the polarity of the polymer, which in turn is responsible for causing greater disorganization of the paraffins. Although none of the materials were able to reduce the PP in the systems containing 6.0 wt/wt % paraffins and 2000 ppm of the active substance (Table 3), this change in the morphology of the crystals seemed to have contributed to the PP reduction capacity presented by EVA11OH in the systems containing 500 and 1000 ppm of the active substance (Table 3).

At 15 °C, EVA11:PALY (Figure 5f1,f2) caused a morphology similar to that of the EVA11 matrix, characterized by long needle-like structures. At 5 °C, a greater spacing of the crystals was observed, likely related to the increase in the degree of polarity of the additive due to the presence of the clay mineral in its composition.

The origin of the increase in polarity in the material, whether by chemical modification in the polymer structure or by the presence of a load in the polymer matrix, can lead to different crystalline morphologies, as observed for EVA11OH and EVA11:PALY (Figure 5e2,f2, respectively).

Similar to EVA11OH, the nanocomposite EVA11OH:PALY (Figure 5g2) also induced a star-shaped morphology. The crystals were formed in a more agglomerated manner, containing a greater amount of wax distributed radially within the crystal structure. Furthermore, a greater spacing between these clusters was observed compared to that of the system containing EVA11OH (Figure 5e2). The crystalline morphology observed in the model system containing EVA11OH:PALY was likely related to the considerable increase in the polarity of the nanocomposite in comparison with the other materials, resulting from both the hydrolysis of the matrix and the incorporation of the clay mineral load in the material structure. This increase in polarity contributed to a more efficient steric hindrance, favoring the flow of the system and ensuring that it was the only material to present a reduction in the PP of the system at 6 wt/wt % paraffin at a concentration of 2000 ppm of active substance (Table 3, Δ_PP_ = −19.5 °C).

Considering that the additives EVA11, EVA11OH, and EVA11:PALY were not able to reduce the PP of the pure model oil when added at a concentration of 2000 ppm of the active substance, the amount of crystals generated simultaneously in the system may have been the determining factor for the interruption of the flow around the PP temperature of the pure system. On the other hand, the lack of compaction of the crystalline mesh, combined with the spacing between the crystals, may have been responsible for the PP reduction capacity observed for these materials when mixed with active substance concentrations below 2000 ppm in the model oils (Table 3). An explanation for this behavior could be related to the lower concentration of additive in the system, which resulted in the formation of fewer nucleation sites, promoting an even greater separation of the crystals and consequently ensuring the flow of the system, as observed in the PP tests. In order to investigate this hypothesis, we carried out POM analyses of model oils with 6.0 wt/wt % paraffin, adding 500 ppm of EVA11, EVA11OH, EVA11:PALY, and EVA11OH:PALY, as shown in Figure 6.

**Figure 6:**
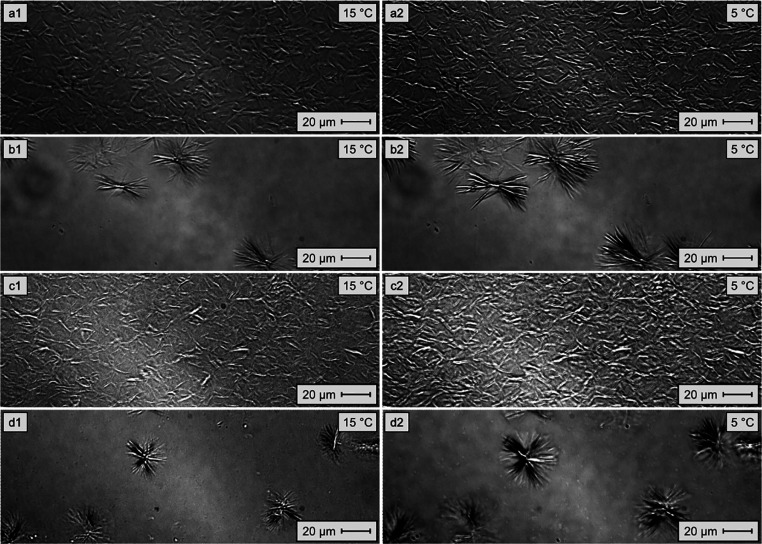
Micrographs of model oils with 6.0 wt/wt % paraffin in toluene, pure and mixed with 500 ppm of the active substance at 15 and 5 °C, respectively: (a1,a2) EVA11; (b1,b2) EVA11OH; (c1,c2) EVA11:PALY; and (d1,d2) EVA11OH:PALY.

The individual morphological characteristics of each system revealed that a reduction in the additive concentration did indeed promote greater spacing between the crystals compared to the systems containing 2000 ppm of the active substance, supporting the hypothesis raised earlier. This effect was less pronounced in the systems containing EVA11 and EVA11:PALY (Figure 6a2,c2) compared with the systems containing the hydrolyzed EVA-based materials (Figure 6b2,d2). Furthermore, there was a reduction in the crystal size in the system containing EVA11OH:PALY (Figure 6b1,b2) compared to that in the system containing only its matrix (Figure 6d2). These behaviors are consistent with the trend observed in the PP tests (Table 3), where the materials based on EVA11 exhibited a lower PP reduction capacity (only reducing the PP at a concentration of 500 ppm of the active substance), while EVA11OH and its nanocomposite reduced PP at both 500 and 1000 ppm of the active substance, with the performance of the nanocomposite being superior to that of EVA11OH. These results suggest that the spacing of crystallization nuclei plays a more important role in the fluidity of the system than just the reduction in the crystal size.

Figure 7 shows the POM test results with model oils at 9.0 wt/wt % paraffin and 500 ppm of the active substance of EVA11, EVA11OH, EVA11:PALY, and EVA11OH:PALY. The systems containing EVA11 and EVA11:PALY (Figure 7a1,a2,c1,c2) keep following the same trend observed in the tests with model oil at 6.0 wt/wt % paraffin, presenting a needle-like morphology with an even more compacted crystalline mesh. This behavior explains the inefficiency of these two materials in reducing the PP of systems with a high paraffin concentration. On the other hand, the model oils containing EVA11OH and EVA11OH:PALY (Figure 7b1,b2,d1,d2) presented a combined morphology, containing the star-shaped crystallization nuclei observed in the systems at 6.0% w/w paraffins and long needle-shaped crystals distributed in different planes. This behavior was likely related to the high concentration of paraffins in the system combined with the low concentration of additive. After the interaction of all the additive available in the system with the paraffins, forming the star-shaped nuclei, the excess wax crystals in the oil were free to maintain their long needle-like morphology, as observed. Despite this combined morphology, once again the star-shaped nuclei exhibited a certain degree of corresponding spacing, which ensured the fluidity of the systems, as observed in the PP tests (Table 3). These results further highlight the importance of crystal spacing for the fluidity of the model oil.

**Figure 7:**
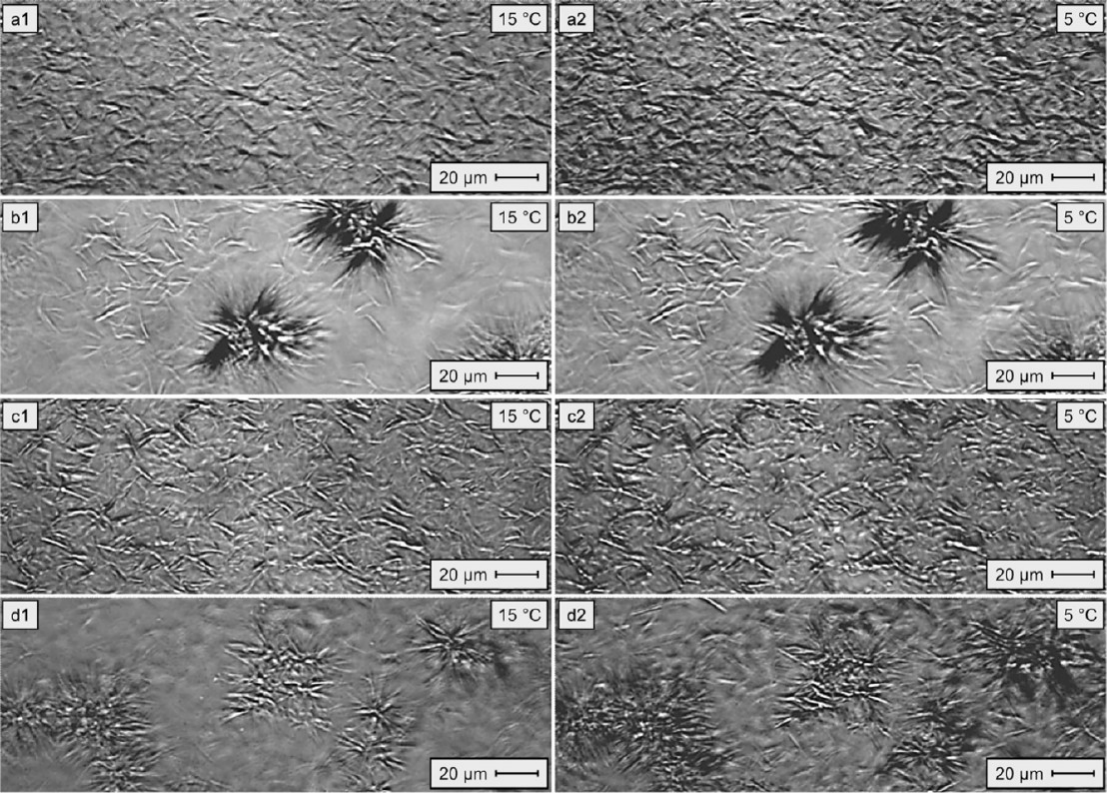
Micrographs of model oils with 9.0 wt/wt % paraffin in toluene, pure and mixed with 500 ppm of active substance at 15 and 5 °C, respectively: (a1,a2) EVA11; (b1,b2) EVA11OH; (c1,c2) EVA11:PALY; and (d1,d2) EVA11OH:PALY.

### 3.5 Correlation between Additive Polarity and Its Mechanism of Action as PPD

PPD additives demonstrated the ability to modify the crystal morphology of the model system through both nucleation and cocrystallization mechanisms, both already presented in the literature.^33,40^ The additives EVA7, EVA11, and EVA11:PALY induced crystal modification through cocrystallization. EVA7OH also presented the same mechanism; however, the presence of void regions between the crystals suggests the influence of additional factors on the modification of the crystal morphology. In contrast, the additives EVA11OH and EVA11OH:PALY acted predominantly by nucleation, with the greatest morphological disorder being observed in the crystals formed in the presence of EVA11OH.

Considering that the additives based on hydrolyzed EVA promoted significant variations in the crystalline morphologies compared to the systems induced by cocrystallization, that the different hydrolyzed samples provoked different crystal morphologies, and that these hydrolyzed additives present significant differences in polarity among them (EVA7OH < EVA11OH < EVA11OH:PALY), it is possible to assume that such behavior is related to the variation in the solubility of these additives in the model system, which has already been demonstrated under other conditions.^39^

Figure 8 proposes an explanation of how the solubility and polarity of materials could influence the crystallization mechanism of waxes. EVA7OH (Figure 8a) tends to have moderate solubility because it has a smaller number of polar groups in its structure. The relative expansion of polymer chains in solution would facilitate the interaction between waxes and ethylene segments, generating cocrystallization sites. Simultaneously, the presence of –OH groups would generate sufficient repulsions between two or more polymer chains, resulting in the void regions observed between crystals (see the micrographs in Figure 5c1,c2). In turn, EVA11OH (Figure 8b) has a greater amount of polar groups, which favors the contraction of the polymer chains in toluene containing waxes. This compact conformation would induce the formation of crystallization nuclei, leading to crystals with a star-shaped morphology. In addition, ethylene segments along the polymer chain could induce cocrystallization, which would explain the morphological disorder observed in micrographs (Figure 5e1,e2). In the case of the EVA11OH:PALY nanocomposite (Figure 8c), besides the high polarity of the material, the compatibilization between the clay mineral and polymer, promoted by CTAB, would be responsible for the formation of intra- and intermolecular interaction bridges among polymer chains. These interactions would not only induce greater contraction of the chains but would also promote their entanglement. The condensed nuclei formed from this entanglement would induce the radial growth of the crystals, resulting in the experimentally observed star-shaped structures (micrographs in Figure 5g1,g2). Because the clay mineral is less soluble in the model system, it would be placed inside the entanglements, not being available to interact with waxes, as observed in the pour point tests at 9.0 wt/wt % paraffin (Table 3).

**Figure 8:**
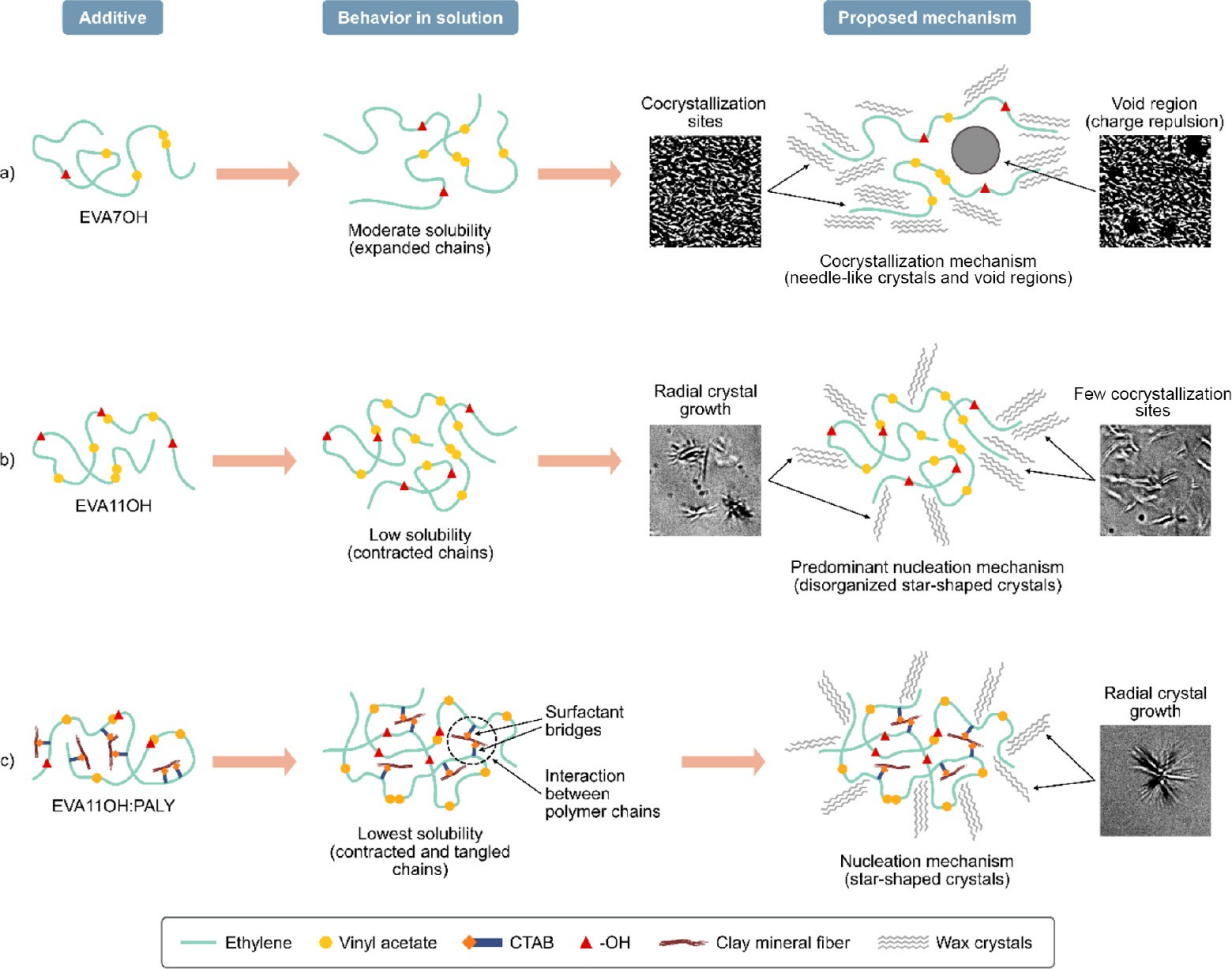
Influence of material polarity/solubility on wax crystallization mechanisms.

## 4 Conclusion

Combined SEM, EDS, and TEM analyses confirmed that the PALY clay mineral were disaggregated and dispersed in the polymer matrix with fiber diameters on a nanometric scale, allowing the classification of the produced materials as nanocomposites.

Unlike the EVA7 and EVA7OH, which showed some performance only at 3.0 wt/wt % waxes, the EVA11, EVA11OH samples, and their respective nanocomposites demonstrated a PPD reduction efficiency even at higher wax concentrations. It seems that only increasing the relative polarity of the molecules, without increasing the number of polar groups in the molecular chains, does not improve the performance.

Among the materials based on EVA11, only EVA11OH and EVA11OH:PALY were able to reduce the PP, from 18.0 °C to <−24.0 °C, in the model oil containing 6.0 wt/wt % paraffin. This reduction was observed with 500 ppm of EVA11OH and 500 and 1000 ppm of EVA11OH:PALY. In waxy model oils containing 9.0 wt/wt % paraffin, these two materials maintained the ability to reduce the PP, although with a weaker performance (Δ_PP_ = −15.0 and −18.0 °C, for EVA11OH and EVA11OH:PALY, respectively), and only at 500 ppm.

All materials modified the morphology of wax crystals but in a different way: EVA7, EVA7OH, EVA11, and EVA11:PALY significantly reduced the size of wax crystals acting by the cocrystallization mechanism and contributing to the formation of a dense and compact crystalline mesh; EVA11OH and EVA11OH:PALY acted by the nucleation mechanism, favoring the formation of a star-shaped morphology with waxes organized radially around the crystallization nucleus. Besides the reduction in the crystal size, the spacing between them plays a crucial role in the fluidity of the system. Similar performances of EVA11OH and EVA11OH:PALY were attributed to the overlapping effect of the hydroxyl groups on the clay mineral.

The distinguishing performances of all samples were associated with their polarity/solubility variations, making it possible to propose different mechanisms of action.

Other important contributions can be made to this research field by investigating the influence of polymer molar mass on its solubility, performance, and mechanism of action.
